# Influence of Extended Itraconazole Antifungal Prophylaxis on the Development of Fungal Infections After Lung Transplant

**DOI:** 10.1155/joot/6995822

**Published:** 2026-02-26

**Authors:** Sabrina Fischer, Raksha Patel, Mufaddal Mamawala, Susan K. Mathai, Katherine Vandervest, Tiana Endicott-Yazdani, Chetan Naik, Todd Grazia, Lisa Fuller

**Affiliations:** ^1^ Department of Pharmacy, Mayo Clinic, Rochester, Minnesota, USA, mayo.edu; ^2^ Department of Pharmacy, Baylor University Medical Center, Dallas, Texas, USA, baylor.edu; ^3^ Department of Biostatistics, Baylor University Medical Center, Dallas, Texas, USA, baylor.edu; ^4^ The Center for Advanced Lung Disease, Baylor University Medical Center, Dallas, Texas, USA, baylor.edu; ^5^ Department of Medicine, Texas A&M University College of Medicine, Dallas, Texas, USA, tamhsc.edu

**Keywords:** amphotericin, antifungal prophylaxis, invasive fungal infection, itraconazole, lung transplant

## Abstract

**Background:**

Invasive fungal infections (IFIs) are associated with a high mortality in lung transplant recipients, with no consensus on optimal antifungal prophylaxis. We aimed to assess the efficacy of long‐term itraconazole compared to short‐term inhaled amphotericin to prevent IFIs post‐transplant.

**Methods:**

A retrospective review of adult lung transplant recipients from January 2016 to September 2022 was conducted. The cohort was divided into two groups based on initial mold prophylaxis: long‐term itraconazole and short‐term inhaled amphotericin. The primary outcome was the incidence of IFIs. The secondary outcomes included the time to IFI, incidence of fungal species found on cultures, and safety/tolerability.

**Results:**

A total of 203 patients met the inclusion criteria (amphotericin group *n* = 108, itraconazole group *n* = 95). The overall incidence of IFIs was significantly higher in the amphotericin group than the itraconazole group (76.9% vs. 56.8%, *p* = 0.002). The Kaplan–Meier curve for the risk of IFI within 1 year of transplant showed a shorter time to IFI in the amphotericin group (*p* = 0.009). In the amphotericin group, there was an increased incidence of positive fungal cultures compared to the itraconazole group with *Aspergillus spp.* (25% vs. 8.4%, *p* = 0.002), *Penicillium spp*. (25.9% vs. 9.5%, *p* = 0.002), yeast (70.4% vs. 36.8%, *p* ≤ 0.001), and other positive fungal cultures (28.7% vs. 12.6%, respectively, *p* = 0.005). The amphotericin group had more discontinuations due to intolerance than the itraconazole group (12% vs. 3.2%, *p* = 0.019).

**Conclusion:**

In adult lung transplant recipients, long‐term prophylaxis with itraconazole was more effective at preventing overall IFIs, positive cultures with *Aspergillus spp.*, and was better tolerated than short‐term inhaled amphotericin.

## 1. Introduction

Lung transplantation can be a lifesaving treatment for patients suffering from advanced lung diseases not responsive to medical treatment modalities. The most common indications include interstitial lung disease (ILD), bronchiectasis, chronic obstructive pulmonary disease (COPD), and pulmonary arterial hypertension (PAH) [1]. Current data reveal the 5‐year survival rate after lung transplant to be 52.7% in males and 58.3% in females [2]. Invasive fungal infections (IFIs) contribute significantly to the mortality seen in lung transplant recipients, with the most common IFIs post–lung transplantation being *Aspergillus spp*., *Candida spp*., and endemic mycoses, which are notably more prevalent in the first year post‐transplantation [3].

Antifungal prophylaxis is routinely administered post‐transplant; however, there is no consensus regarding the optimal antifungal agent or duration of use [3, 4]. The American Society of Transplantation Infectious Diseases Community of Practice Guidelines for Invasive Aspergillosis in solid‐organ transplant recipients recommends antifungal prophylaxis regimens active against *Aspergillus spp.* for lung transplant recipients, including itraconazole, voriconazole, posaconazole, isavuconazole, or inhaled amphotericin B [5]. The International Society for Heart and Lung Transplantation recommends including coverage for *Candida spp.* for up to 90 days after pulmonary transplant and extending mold‐active prophylaxis for up to 180 days after surgery [6]. In a 2019 worldwide study of lung transplantation centers, most centers utilized a universal post‐transplant prophylaxis regimen of inhaled amphotericin with a systemic triazole (most commonly itraconazole) for 3–6 months [7].

Previous studies found that the implementation of an antifungal prophylaxis regimen reduces the incidence of IFIs [8, 9]. However, there are limited data comparing the safety and efficacy of the individual regimens. One study demonstrating a difference between antifungal regimens revealed that posaconazole was more effective at preventing IFIs than itraconazole when utilized short‐term in critically ill lung transplant recipients in their immediate postoperative period [10]. Prior studies comparing voriconazole to either itraconazole or isavuconazole found no difference in IFIs but did reveal an increase in hepatotoxicity in the voriconazole group [11–13]. However, when patients with a baseline increased liver risk were stratified to receive posaconazole, there was no difference in hepatotoxicity or IFI rate observed between the posaconazole and voriconazole groups [14].

This study seeks to add to the current literature comparing the efficacy and safety of antifungal prophylaxis regimens for adult lung transplant recipients. Our institutional protocol historically utilized inhaled amphotericin B post‐transplant during their index hospital admission (for 30 days or discharge), followed by nystatin upon discharge. The protocol was changed in May 2019 to use itraconazole 100 mg BID for at least one year post‐transplant. The primary objective of this study was to evaluate the incidence of IFIs within one year post‐transplant. The secondary outcomes were to evaluate the incidence of and time to the development of IFI, positive fungal cultures, mortality, and the safety and tolerability of each regimen.

## 2. Materials and Methods

### 2.1. Patients and Setting

This IRB‐approved retrospective study was carried out at Baylor University Medical Center in Dallas, Texas, a 1008‐bed nonprofit academic medical center with a comprehensive lung transplant program and approved by the institutional IRB. All adult patients who received a single or bilateral lung transplant between January 2016 and September 2022 were screened for inclusion in the study. Patients were excluded from the study if they received antifungal prophylaxis off‐protocol, received antifungal treatment within the 24 h prior to transplant, were recipients of multiorgan transplant, had a previous organ transplant, expired within 7 days of transplant, or were taking medication that is contraindicated with itraconazole. Data were collected for 1 year post‐transplant. Patients received a standard maintenance immunosuppression consisting of tacrolimus immediate release (initial goal trough 10–14 ng/mL with consideration of a lower goal of 8–12 ng/mL for estimated CrCl ≤ 60 mL/min or age ≥ 65), mycophenolate mofetil 1000 mg BID, and a prednisone taper. Basiliximab represented the primary induction agent. All IFI diagnoses met definitions for any proven, probable, and possible IFIs according to the European Organization for Research and Treatment of Cancer/Mycoses Study Group Education and Research Consortium (EORTC/MSGERC) guidelines [15].

### 2.2. Study Population

Our institutional protocol historically utilized short‐term mold prophylaxis (inhaled amphotericin deoxycholate or liposomal 25 mg twice daily for 30 days or until discharge with or without extension with nystatin or fluconazole) during their index hospital admission. The protocol was changed in May 2019 to use long‐term mold prophylaxis with itraconazole 100 mg by mouth twice daily for at least one year with an indefinite extension if tolerated and affordable. Patients were grouped by prophylaxis: inhaled amphotericin before, or itraconazole after, the protocol change. Itraconazole was selected as the antifungal agent of choice due to cost and tolerability. Historically, our institution has seen improved medication access due to lower cost when utilizing prophylactic itraconazole and reserving other azole antifungals for treatment of infection. Itraconazole has variable absorption and bioavailability, with differences cited between formulations, food intake, and gastric acidity. Although prolonged prophylaxis with azole antifungals may warrant therapeutic drug monitoring, there are no randomized controlled trials guiding the optimal target itraconazole levels for prophylaxis with varying guidance only through observational studies and meta‐analyses [16, 17]. Therefore, therapeutic drug monitoring for itraconazole was not routinely performed when used for prophylaxis at our institution. Although SUBA®‐itraconazole became available in 2018 with a greater bioavailability, all formulations of itraconazole utilized in this study consisted of conventional itraconazole solution with transition to capsules once able to take medications orally [18].

### 2.3. Statistical Methods

Data were analyzed using SAS v9.4. A chi‐square or Fisher’s exact test was used to evaluate categorical data as appropriate, and a Student’s *t*‐test was used to evaluate continuous data. Secondary outcomes of the risk of IFI and mortality were evaluated by using Kaplan–Meier survival curves with a log‐rank test to evaluate statistical significance. The survival free of IFI within the Kaplan–Meier analysis was further evaluated with a Wilcoxon signed rank test. A Cox proportional hazards model for the risk of the development of IFI was performed for baseline characteristics with statistical significance (excluding variables with sample sizes too small for calculations). Pre‐existing or donor‐related cultures were excluded from the overall IFI analysis to help differentiate between a true IFI and colonization or infection not related to the selection of the antifungal prophylaxis agent.

## 3. Results

### 3.1. Patient Characteristics

A total of 238 patients were screened with 203 patients meeting the inclusion criteria (amphotericin group *n* = 108, itraconazole group *n* = 95). In the itraconazole group, there was a greater number of patients of Hispanic ethnicity (13.9% vs. 1.9%, *p* = 0.001) and fewer listing indications for cystic fibrosis (1.05% vs. 8.33%, *p* = 0.021). All other baseline characteristics were similar between the two groups (Table 1). The median duration (range) of therapy for inhaled amphotericin was 14 (9–30) days in the amphotericin group. For immunosuppression, the median dose of tacrolimus was lower in the itraconazole group at 12 months (1.5 vs. 3.5 mg, *p* < 0.001). Fewer patients in the itraconazole group received treatment for rejection in the first year post‐transplant (36.9% vs. 53.7%, *p* = 0.016) and received broad‐spectrum antibiotic therapy for 5–7 days within 90 days prior to IFI (30.7% vs. 56.6%, *p* < 0.001). In comparison to the amphotericin cohort during the first year post‐transplant, the itraconazole group had a greater number of patients develop hypogammaglobulinemia (25.3% vs. 12.5%, *p* = 0.025), as well as more necrotic mucosal slough (24.1% vs. 5.6%, *p* < 0.001), and was less likely to develop viral infections (38.8% vs. 47.2%, *p* = 0.018) or neoplasms (21.1% vs. 34.3%, *p* = 0.037) after transplant (Table 1).

**TABLE 1:** Baseline characteristics and risk factors for IFI.

Characteristic	Itraconazole group *n* = 95	Amphotericin group *n* = 108	*p*‐value
Age at transplant; median (IQR)	63 (56–66)	62 (56.5–65.5)	0.660
Height; median (IQR)	172.7 (162.6–177.8)	172.7 (162.6–177.8)	0.700
Weight; median (IQR)	78.5 (67.2–90.5)	76.9 (65.3–88.5)	0.425
BMI; median (IQR)	26.9 (23.5–30.3)	26.5 (23.5–28.9)	0.173
Type of transplant			
Bilateral	69 (72.63%)	66 (61.11%)	0.101
Single left	15 (15.79%)	21 (19.44%)	0.582
Single right	11 (11.58%)	21 (19.44%)	0.176
Primary listing indication for lung transplant			
IPF	42 (44.21%)	46 (42.59%)	0.887
Non‐IPF ILD	2 (2.11%)	1 (0.93%)	0.600
COPD	26 (27.37%)	34 (31.48%)	0.541
A1AT‐related COPD (alpha‐1 anti‐trypsin deficiency)	3 (3.16%)	3 (2.78%)	1
PAH	2 (2.11%)	1 (0.93%)	0.604
CF (cystic fibrosis)	1 (1.05%)	9 (8.33%)	0.021
Bronchiolitis	1 (1.05%)	1 (0.93%)	1
Bronchiectasis (non‐CF)	2 (2.11%)	1 (0.93%)	0.600
Other	17 (17.89%)	12 (11.11%)	0.228
Gender			0.718
Male	53 (55.79%)	63 (58.33%)	
Female	41 (43.16%)	45 (41.67%)	
Other	1 (1.05%)	0	
Race			
White	86 (90.53%)	95 (87.96%)	0.653
Black or African American	5 (5.26%)	11 (10.19%)	0.297
Asian	3 (3.16%)	1 (0.93%)	0.342
American Indian/Alaska Native	1 (1.05%)	1 (0.93%)	1
Ethnicity			0.001
Hispanic or Latino	13 (13.68%)	2 (1.85%)	
Not Hispanic or Latino	82 (86.32%)	106 (98.15%)	
Comorbidities			0.375
Diabetes mellitus	3 (3.16%)	6 (5.56%)	
HFpEF	1 (0.05%)	1 (0.93%)	
HFrEF	1 (1.05%)	0	
Coronary artery disease	18 (18.95%)	12 (11.11%)	
Chronic kidney disease	0	2 (1.85%)	
Liver disease	3 (3.16%)	7 (6.48%)	
On immunosuppression at the time of transplant	22 (23.16%)	22 (20.37%)	
Sinus disease	5 (5.26%)	7 (6.48%)	
HIV/AIDS	1 (1.05%)	0	
Hypogammaglobulinemia	2 (2.11%)	0	
None	39 (41.05%)	51 (47.22%)	
Tacrolimus dose at 12 months post‐transplant	1.5 (1–3)	3.5 (2–5)	< 0.001
Received treatment for rejection within 12 months of transplant	35 (36.84%)	58 (53.70%)	0.016
Broad‐spectrum antibiotic therapy for 5–7 days within 90 days prior to IFI	23 (30.67%)	56 (56.57%)	< 0.001
Incidence of viral infections	35 (36.84%)	51 (47.22%)	0.018
Necrotic mucosal slough reported in the first 12 months post‐transplant	23 (24.21%)	6 (5.56%)	< 0.001
Change in ejection fraction from baseline; mean (SD)	7 (5.69)	6.09 (5.45)	0.329
Inhaled amphotericin duration of therapy (days) (IQR)	8 (0–11)	14 (9–30)	< 0.001
Intolerant of antifungal prophylaxis, requiring early discontinuation	3 (3.2%)	13 (12%)	0.019
Change in diastolic grade from baseline; mean (SD)	1.64 (1.92)	1.69 (1.88)	0.873
12 mo. post‐transplant AST; mean (SD)	24.71 (15.07)	21.67 (11.81)	0.132
12 mo. post‐transplant ALT; mean (SD)	26.08 (22.78)	29.17 (25.10)	0.242
12 mo. post‐transplant T bilirubin; mean (SD)	0.44 (0.23)	0.48 (0.22)	0.238
SCr value at the time of transplant (pretransplant, in mg/dL); median (IQR)	0.75 (0.65–0.92)	0.86 (0.72–0.98)	0.008
12 mo. post‐transplant serum creatinine (mg/dL); median (IQR)	1.20 (1.02–1.48)	1.31 (1.13–1.61)	0.032

### 3.2. IFIs

**FIGURE 1:**
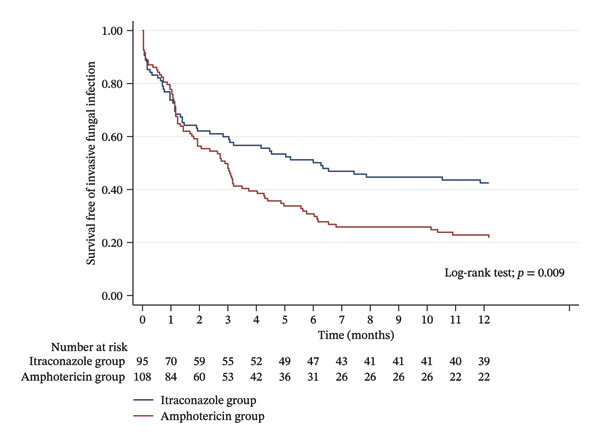
Time to IFI among post–lung transplant patients.

The overall incidence of IFIs (not pre‐existing or donor‐related) was significantly higher in the amphotericin group than the itraconazole group in the first year post‐transplant (76.9% vs. 56.8%, *p* = 0.002) (Table 2). All positive cultures were included in subsequent analyses unless otherwise stated including infections that were previously identified as pre‐existing or donor‐related. Of all IFIs, 16% met EORTC/MSGERC definitions for proven IFI and 84% met criteria for probable IFI in the two groups. The Kaplan–Meier curve for the risk of IFI within 1 year of transplant showed a shorter time to IFI in the amphotericin group (*p* = 0.009), and this graph was developed from the overall incidence of IFIs (Figure 1). The survival estimates from the Kaplan–Meier analysis for the risk of IFI were further delineated to show that the risk of IFI begins to differentiate around 45 days post‐transplant (*p* = 0.009 by log rank, *p* = 0.08 by Wilcoxon) (Table 3). We note that the log‐rank test yielded a statistically significant result, whereas the Wilcoxon test did not. This difference is due to the distinct weighting schemes inherent to the two procedures. The log‐rank test applies equal weight across all events and is therefore most sensitive to differences in survival that arise in the later portion of the survival curve. In contrast, the Wilcoxon test assigns greater weight to early failures and consequently emphasizes early differences in survival. The above data suggest that earlier rates of survival free of IFI were similar between the two arms, with between‐group differences occurring predominantly during the later follow‐up period (post‐45‐day mark as seen in Table 3).

**TABLE 2:** Incidence of IFIs.

	Itraconazole group	Amphotericin group	*p*‐value
Overall IFIs	54 (56.8%)	83 (76.9%)	0.002

**TABLE 3:** Survival estimates from Kaplan–Meier analysis for risk of IFI development.

Time since transplant (days)	Survival free of IFI	
	Itraconazole (%)	Amphotericin B (%)
5	85.3	87.0
10	83.2	86.1
15	82.1	85.2
20	79.0	82.4
25	76.8	79.6
30	73.7	77.8
35	68.4	67.6
40	67.4	63.9
45	64.2	62.0
60	61.03	55.4
90	58.9	47.9
180	50.1	29.8
270	44.7	25.9
300	44.7	24.9
330	43.6	22.8
365	42.5	21.8
Test	*p* ‐ value	
Log‐rank	0.009	
Wilcoxon	0.080	

The proportional hazards assumption for the itraconazole and amphotericin arms was not satisfied (supremum test for proportional hazards assumption; *p* = 0.038), so we fitted a piecewise multivariable Cox proportional hazards model including a time‐dependent interaction term, with a change point at 45 days (based on Table 3), which allows for varying hazards over time [19]. The baseline hazard for itraconazole relative to amphotericin was not statistically significant (HR = 1.263, 95% CI 0.607–2.627, *p* = 0.533), indicating no significant difference in the risk of IFI development during the early follow‐up period (up to 45 days). However, the interaction term was significant (HR = 0.384, 95% CI 0.182–0.819; *p* = 0.010), demonstrating that the effect of itraconazole on risk of IFI changes significantly over time, with a progressively lower hazard relative to the amphotericin group after 45 days. Post‐45‐day HR is derived from the sum of the main effect (treatment) and the interaction term. The post‐45‐day HR for itraconazole versus amphotericin was 0.484, 95% CI 0.220–1.067; *p* = 0.072 (Table 4). It should be noted that combining two coefficient estimates increases the variance of the combined estimates because the standard errors of both coefficients contribute to the total variance, thereby leading to wider confidence intervals and higher *p*‐values. Despite the post‐45‐day HR for itraconazole not reaching statistical significance, the significant interaction term indicates a robust time‐dependent effect. Thus, it is reasonable to assume that the combination of two coefficients that produce the post‐45‐day HR, along with a relatively lower number of events, contributed to the nonsignificant *p*‐values, rather than indicating an absence of a true effect. Among other covariates, only recent broad‐spectrum antibiotic therapy within 90 days prior to IFI was significantly associated with increased risk (HR = 1.822, 95% CI 1.254–2.606; *p* = 0.001), while other risk factors were not statistically significant (Table 4).

**TABLE 4:** Cox proportional hazards model for the risk of development of IFI.

Variable	Hazard ratio	95% confidence interval	*p*‐value
Itraconazole vs. amphotericin at baseline	1.263	0.607–2.627	0.533
Itraconazole arm × post‐45 days (interaction)^∗^	0.384	0.182–0.819	0.010
Necrotic mucosal slough reported in the first 12 months post‐transplant (Yes vs. No)	1.284	0.771–2.136	0.337
Neoplasms (Yes vs. No)	0.751	0.523–1.141	0.152
Received treatment for rejection at 12 months post‐transplant (Yes vs. No)	1.224	0.854–1.756	0.278
Broad‐spectrum antibiotic therapy for 5–7 days within 90 days prior to IFI (Yes vs. No)	1.822	1.254–2.606	0.001
Viral infections (Yes vs. No)	0.910	0.629–1.267	0.598
Year of transplant (per‐year increase)	0.972	0.817–1.166	0.760

^∗^This hazard ratio represents the interaction between the itraconazole and amphotericin groups and time.

### 3.3. Positive Fungal Cultures

In the amphotericin group, there was an increased incidence of positive cultures compared to the itraconazole group with all *Aspergillus spp.* (25% vs. 8.4%, *p* = 0.002), *Aspergillus flavus* (6.5% vs. 2.1%, *p* = 0.015), *Aspergillus spp.* (other) (10.2% vs. 2.1%, *p* = 0.019), *Penicillium spp.* (25.9% vs. 9.5%, *p* = 0.002), undifferentiated yeast (70.4% vs. 36.8%, *p* < 0.001), and other IFI (28.7% vs. 12.6%, *p* = 0.005), respectively. In the itraconazole group, there was an increased incidence of overall *Candida spp.* (57.9% vs. 36.1%, *p* = 0.002). There was no difference between the two groups regarding the incidence of other positive cultures such as *Aspergillus fumigatus, Histoplasma spp., and z*ygomycetes (*Mucor spp*.). The itraconazole group had more fungal cultures sourced from donor swabs (52.6% vs. 26.3%, *p* < 0.001) and fewer fungal cultures sourced from BALs (43.4% vs. 64.7% *p* = 0.006) (Table 5). Patients in the itraconazole group had a smaller number of positive fungal cultures within 12 months post‐transplant (80% vs. 91.7%, *p* = 0.016) (Table 5).

**TABLE 5:** Incidence of positive fungal cultures.

Type of fungus	Itraconazole group	Amphotericin group	*p*‐value
Any *Aspergillus spp.*	8 (8.4%)	27 (25%)	0.002
* Aspergillus fumigatus*	2 (2.1%)	5 (4.6%)	0.452
* Aspergillus flavus*	0 (0%)	7 (6.5%)	0.015
* Aspergillus terreus*	2 (2.1%)	1 (0.93%)	0.600
* Aspergillus niger*	3 (3.2%)	5 (2.6%)	0.726
Undifferentiated *Aspergillus spp.*	2 (2.1%)	11 (10.2%)	0.019
Any *Candida spp.*	55 (57.9%)	39 (36.1%)	0.002
* Candida albicans*	34 (35.8%)	29 (26.9%)	0.170
* Candida glabrata*	7 (7.4%)	3 (2.8%)	0.194
* Candida krusei*	3 (3.2%)	0 (0%)	0.101
* Candida tropicalis*	6 (6.3%)	3 (2.8%)	0.310
Undifferentiated *Candida spp.*	19 (20%)	9 (8.3%)	0.016
*Cryptococcus spp.*	1 (1.05%)	4 (3.7%)	0.374
*Coccidioides spp.*	0 (0%)	1 (0.93%)	1.00
*Histoplasma spp.*	0 (0%)	0 (0%)	N/A
*Fusarium spp.*	0 (0%)	2 (1.85%)	0.500
*Penicillium spp.*	9 (9.5%)	28 (25.9%)	0.002
*Scedosporium spp.*	0 (0%)	0 (0%)	N/A
Zygomycetes *(Mucor spp.)*	0 (0%)	0 (0%)	N/A
Undifferentiated yeast	35 (36.8%)	76 (70.4%)	< 0.001
Other	12 (12.6%)	31 (28.7%)	0.005
Positive fungal culture	76 (80.0%)	99 (91.67%)	0.016
Positive beta‐D‐glucan	12 (30.77%)	25 (44.64%)	0.173
Positive *Aspergillus* galactomannan from any source	6 (11.76%)	3 (6.0%)	0.309
Positive *Aspergillus* galactomannan in BAL Positive *Aspergillus* galactomannan in plasma/serum	5 (83.33%) 1 (16.67%)	1 (33.33%) 2 (66.67%)	0.226
Source of fungal culture			
Donor swab	40 (52.63%)	26 (26.26%)	< 0.001
BAL	33 (43.42%)	64 (64.65%)	0.006
Sputum	1 (1.32%)	0	0.434
Pleural	0	3 (3.03%)	0.259
Blood	0	3 (3.03%)	0.259
Other	2 (2.63%)	3 (3.03%)	1

### 3.4. Safety and Tolerability

**FIGURE 2:**
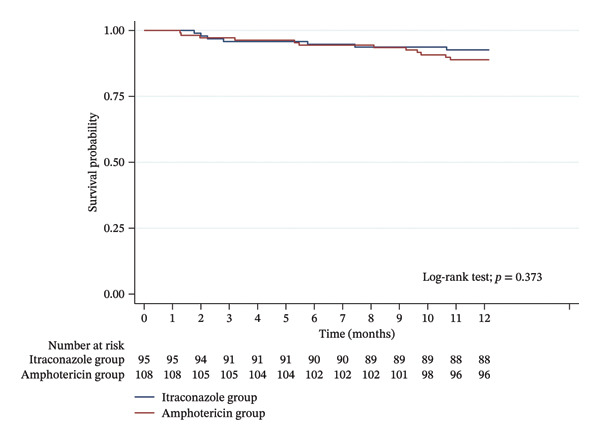
Risk of mortality among post–lung transplant patients.

The amphotericin group had increased levels of intolerance compared to the itraconazole group (12% vs. 3.2%, *p* = 0.019), primarily due to bronchospasm or nausea requiring discontinuation of therapy. Those who discontinued inhaled amphotericin either received nystatin or no prophylaxis. There was no statistical difference in liver function tests and the itraconazole group had a lower serum creatinine at 12 months post‐transplant (1.2 vs. 1.31 mg/dL, *p* = 0.032), but notably also had a lower serum creatinine at baseline (0.75 vs. 0.86 mg/dL, *p* = 0.008). On echocardiogram, there were no differences in ejection fraction or diastolic grade between the two groups (Table 1). The Kaplan–Meier survival test for the 1‐year incidence of mortality showed no difference between the two groups (*p* = 0.373) (Figure 2).

## 4. Discussion

This study investigated the difference in the incidence of IFIs in adult lung transplant recipients who received either short‐term inhaled amphotericin or long‐term itraconazole antifungal prophylaxis. Neoh et al. found that azole antifungal prophylaxis resulted in a low incidence of IFI and IFI‐related mortality at 12 months post‐transplant but did not have a comparator control group [8]. Our study found a similar result in a lower incidence of IFI with the itraconazole group when compared to a historical control group utilizing short‐term inhaled amphotericin. Although our rates of IFI were higher than the reported literature, only 16% of IFIs met EORTC/MSGERC criteria for proven IFI, and not all IFIs were clinically significant. Our study did not find a significant difference in overall mortality but did not differentiate the cause of mortality to assess whether death was related to IFI, since our study was not powered to assess this result. Reichenspurner et al. found that the use of inhaled amphotericin during the index admission compared to a control group that did not receive any antifungal prophylaxis decreases overall IFIs at 12 months post‐transplant [9]. Our study similarly saw that inhaled amphotericin decreased IFIs, but once mold‐active therapy was discontinued, our long‐term prophylaxis group was more effective at preventing IFIs. However, the use of a long‐term oral agent with *Aspergillus spp.* coverage has become increasingly utilized in a recent survey [7]. Samata et al. compared isavuconazole and voriconazole for a median of 3 months and saw no difference between the two agents, but as mold‐active prophylaxis was discontinued, the number of mold infections increased [12]. Our study reflects a similar result with our short‐term amphotericin cohort developing more mold infections compared to our long‐term itraconazole cohort. This difference was seen in our Kaplan–Meier curve assessing time to IFI, where the rate of IFI was similar between the two groups until amphotericin was discontinued at 1 month post‐transplant, reflecting that long‐term mold coverage was more effective at preventing IFIs (Figure 1). When we divided the survival estimates from the Kaplan–Meier analysis, we saw a divergence begin around 45 days post‐transplant (Table 3). In addition, this indicates that the rate of IFIs is only decreased while mold‐active agents are utilized.

Although the rates of IFI were similar while amphotericin was utilized, our patients did not tolerate inhaled amphotericin as well as itraconazole although the at‐risk time of inhaled amphotericin was shorter than itraconazole. Pióro et al. found no difference between itraconazole and voriconazole in the difference of IFIs at 12 months post‐transplant but focused on *Candida spp.* and *Aspergillus spp.*, while our study looked at a broad range of fungal organisms and overall incidence of IFI [10]. This result is similar to our study in that the rate of IFIs is reduced, while mold‐active agents are utilized. We found a similar result as Cadena et al., with no significant hepatotoxicity in our itraconazole‐treated patients in a larger cohort with effective prevention of IFIs. This reflects the outcome that while mold‐active therapies are utilized, the incidence of IFIs decreases [11].

Patient characteristics were similar between the two groups except for ethnicity and primary listing indication for transplant. A greater number of Hispanic patients in the itraconazole group reflects the growing diversity of our transplant patient cohort over time [20]. The introduction of CFTR modulators, particularly elexacaftor–tezacaftor–ivacaftor, has significantly reduced the need for lung transplant in the cystic fibrosis population over time, which is demonstrated in fewer listing indications for cystic fibrosis in the itraconazole group [21]. Both the amphotericin and the itraconazole groups had baseline characteristics associated with an increased risk of IFIs, including an 18% higher risk among patients who received broad‐spectrum antibiotic therapy for 5–7 days within 90 days prior to IFI. The increased amount of necrotic mucosal slough may represent more ischemic injury and decreased localized efficacy of a systemic antifungal agent, such as itraconazole. However, the difference in necrotic mucosal slough may be due to documentation differences between electronic health record (EHR) systems utilized in the two groups. In terms of safety and tolerability, itraconazole has been associated with side effects in new or worsening heart failure or an increase in liver enzymes [18], but this was not observed in our cohort, and itraconazole was better tolerated than inhaled amphotericin.

This study has several limitations. There were many undifferentiated yeast cultures in the amphotericin group that may account for differences seen in *Candida spp.* infections. The incidence of *Pneumocystis jiroveci* pneumonia (PJP) and tacrolimus trough levels was not evaluated in this study. Itraconazole therapeutic drug monitoring was not routinely performed for prophylaxis to help guide dosing related to efficacy and toxicity. Given the known variability in itraconazole bioavailability and acid‐dependent absorption in patients commonly on acid‐reducing medications, systemic exposure to itraconazole was likely variable. Patients received amphotericin or itraconazole during different time periods, and it is possible that ongoing advances in transplantation medicine and surgery, changes in other institutional protocols, and advancements in the EHR systems during the study period influenced our results [22]. Amphotericin formulation changed depending on availability, and data on specific formulation were not collected as they were used interchangeably. We did not stratify our results by extension of nystatin or fluconazole in the inhaled amphotericin group, which may provide additional yeast coverage. While IFIs represent EORTC/MSGERC definitions in our study, this may not represent clinically significant IFIs, especially considering the large number of undifferentiated yeast cultures in our historical amphotericin cohort that were not further defined by our lab. Due to the retrospective single‐center nature of the study, a bias due to unmeasured confounders cannot be excluded. Large, multicenter, prospective randomized controlled trials are needed to confirm these results. A cost‐benefit analysis could be considered for future studies comparing an itraconazole with therapeutic drug monitoring cohort to an itraconazole cohort without therapeutic drug monitoring.

In conclusion, long‐term mold prophylaxis with itraconazole increased the time to IFI, was more effective at preventing overall IFI, positive fungal cultures with *Aspergillus spp., Penicillium spp.*, and yeast and was better tolerated than short‐term inhaled amphotericin with no effect on mortality outcomes in adult lung transplant recipients within the first year of transplant. Our study favors the use of long‐term itraconazole over short‐term inhaled amphotericin for antifungal prophylaxis.

## Funding

No funding was received for this manuscript.

## Disclosure

The authors have nothing to report.

## Conflicts of Interest

The authors declare no conflicts of interest.

## Data Availability

The data that support the findings of this study are available from the corresponding author upon reasonable request.
